# Healthier Communities of Phytoplankton and Bacteria Achieved via the Application of Modified Clay in Shrimp Aquaculture Ponds

**DOI:** 10.3390/ijerph182111569

**Published:** 2021-11-04

**Authors:** Yu Ding, Xiuxian Song, Xihua Cao, Liyan He, Shanshan Liu, Zhiming Yu

**Affiliations:** 1 CAS Key Laboratory of Marine Ecology and Environmental Sciences, Institute of Oceanology, Chinese Academy of Sciences, Qingdao 266071, China; dingyu@qdio.ac.cn (Y.D.); caoxh@qdio.ac.cn (X.C.); hely@qdio.ac.cn (L.H.); liushanshan336@foxmail.com (S.L.); zyu@qdio.ac.cn (Z.Y.); 2 Laboratory of Marine Ecology and Environmental Science, Qingdao National Laboratory for Marine Science and Technology, Qingdao 266237, China; 3 Chinese Academy of Sciences, Beijing 100049, China; 4 Center for Ocean Mega-Science, Chinese Academy of Sciences, Qingdao 266071, China

**Keywords:** phytoplankton, bacteria, community composition, community structure, modified clay, shrimp aquaculture water

## Abstract

The composition and stability of microbial communities in aquaculture water are crucial for the healthy growth of shrimp and present considerable risk to aquatic ecosystems. The modified clay (MC) method has been proposed as an efficient and safe solution for the mitigation of harmful algal blooms (HABs). Currently, the effects of MC on microbial communities in aquaculture water remain unknown. Here, we adopted the MC method to regulate shrimp-culture water quality and evaluated the effects of MC on the composition and stability of phytoplankton together with bacteria communities through high-throughput sequencing. On the one hand, a prominent change in the composition of microbial community was observed, with green algae becoming the most abundant genera and pathogens being infrequent in the MC-treated pond, which was more conducive to the growth of shrimp than that in the control pond. Moreover, MC could increase the diversity and stability of the microbial community structure in the water column, which had a higher anti-interference ability, as demonstrated by the analysis of the diversity and molecular ecological network. Taken together, MC could reduce the possibility for the occurrence of HABs and maintain a stable microbial community, which is beneficial for the health and high yield of shrimp.

## 1. Introduction

Shrimp aquaculture has grown rapidly over several decades to become an economically important global industry [1]. Currently, shrimp aquaculture is generally carried out at high density in order to ensure high yields. However, the high-density and intensive breeding mode produces a large number of unused feed and related metabolites, which enriches the nutrition of aquaculture water and makes it prone to harmful algal blooms (HABs) [2,3], affecting the stability of microbiota structure. Massive HABs have been documented in shrimp culture ponds from different regions of the world, causing significant worldwide economic losses [4]. The outbreak of HABs significantly increases the ammonia concentration in the water, causing shrimp poisoning and death, or secretes mucus and adheres to shrimp gills, resulting in shrimp dyspnea and even suffocation [4,5]. Furthermore, some algal organisms contain toxins, which are easy to accumulate in shrimp hepatopancreas that may affect humans through consumption of the shrimp [6]. Moreover, with excessive nutrients and the fluctuation of phytoplankton community, the imbalance of the bacterial community is becoming another important threat to the sustainable development of the shrimp aquaculture industry [7]. The proliferation of pathogens and the reduction of probiotics can result in disease and massive mortalities of aquaculture shrimp [8,9,10,11,12]. Therefore, a healthy and stable phytoplankton and bacterial ecosystem is an important requirement for the health and high yield of aquaculture organisms. These processes present challenges for water treatment.

The modified clay (MC) method is one of the few methods that can be used for the mitigation of HABs ever applied in the field, with no major risks to the environment at low concentrations [13]. The principle of algae removal by MC is that the MC collides with the HAB cells and causes them to flocculate, so that the HAB cells settle to the bottom layer and die, and they damage the non-flocculated algae cells so as to effectively eliminate HABs [14]. In addition, both laboratory and mesocosm results have demonstrated that MC can adsorb nutrients in the water [15], slow nutrient circulation [16], and reduce toxin content in the water [17], while regulating the phytoplankton community. Recent research shows that MC also has a certain ability to remove bacteria [18], and different types of MC have demonstrated different effects on the structure and function of the bacterial community. Some studies have also shown that clay flocculation can be used to settle bacteria in fish and shrimp aquaculture to prevent the infection of cultured organism larvae by pathogens [19].

To date, the outcomes of MC treatment among microbial communities have scarcely been investigated. To further characterize the effects of MC on microbial communities in a natural environment, we applied the MC method in a shrimp aquaculture pond. We focused on the following two aspects: (i) how the MC affects the microbial community composition and structure, and (ii) whether these effects are advantageous for shrimp growth. Therefore, high-throughput sequencing technology was used to analyze the differences in phytoplankton and bacterial community composition in aquaculture water after regulation by MC. Simultaneously, based on random matrix theory, we constructed phytoplankton and bacterial molecular ecological networks in aquaculture water and analyzed the differences in microbial network structure, species interaction, and key species. Furthermore, we identified the key environmental factors affecting the structure of the microbial community in the water, which can provide theoretical support for a new model of MC technology that leads toward healthy aquaculture.

## 2. Materials and Methods

### 2.1. Experimental Site

**Figure 1:**
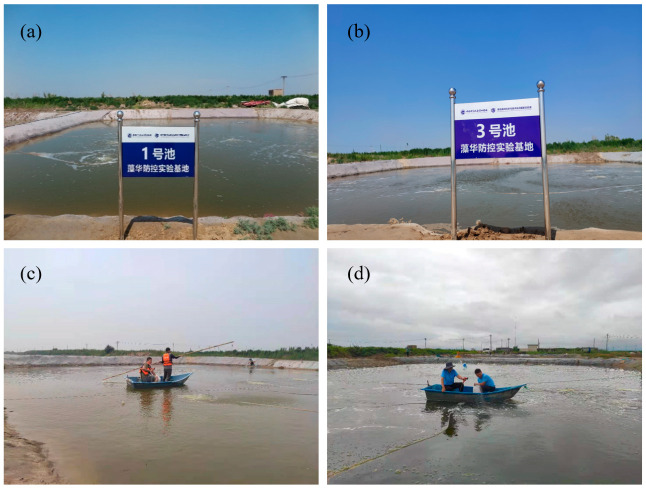
Field photos of control pond (**a**), MC-treated pond (**b**), and on-site operation of MC (**c**,**d**).

The intensive rearing ponds were built in Dongying, Shandong province, Northern China (37°27′ N, 118°54′ E). These ponds were approximately the same size (1000 m^2^) and depth (1.2–1.5 m) (Figure 1a,b), culturing *Litopenaeus vannamei*, shrimp stocking density (50,000 shrimp per pond), feed type, and schedule. The ponds were located outside, and a suitable level of dissolved oxygen was maintained using an impeller aerator. In the control pond, shrimp were cultured in the traditional model. In the experimental pond, we applied MC to control the microbial community and water quality (Figure 1c,d), while the other aquaculture methods were the same as those used in the control pond. Since algal blooms broke out in the control pond in early September 2020, MC was used to eliminate the algal blooms, and the MC technology was also used for regulation during the follow-up. Therefore, we only analyzed the microbial community from 29 June to 13 September in this study. In the following section, the control pond and MC-treated pond are described in detail. In addition, in this paper, the microbial community includes the phytoplankton and bacterial communities.

### 2.2. Preparation of the MC

The experiment clay was natural clay mineral kaolin, obtained from Yankuang Beihai Kaolin Co., Ltd. (Beihai, China). The elemental composition and content of the clay were determined by XRF, as shown in Table S1. Due to the low content of heavy metals (Table S1) and the low content of bacteria carried by itself [18] in clay, their impact on microbial community would not be considered in this paper. The MC used in this study was based on polyaluminum chloride and aluminum sulfate modified clays, termed MCI and MCII, respectively [20,21]. In order to better use MC for intervention, we monitored the phytoplankton concentration every day through tracking sampling. According to the algae composition and water quality characteristics of the aquaculture water on site, we chose different MC to treat. The type, dosage, and treatment time of MC are shown in Table S2.

### 2.3. Sample Collection

In this study, samples were collected 12 times during shrimp aquaculture, which were 29 June, 30 June, 3 July, 24 July, 28 July, 18 August, 26 August, 2 September, 6 September, 9 September, and 13 September. The water samples were collected at approximately 50 cm below the water surface and then transported back to the laboratory in 30 min for analysis of physical and chemical parameters. Field observation and sampling indicators included temperature (T), salinity (S), pH, turbidity (TUR), dissolved oxygen (DO), and nutrient content, including total ammonia nitrogen (TAN, including non-ionic ammonia (NH_3_) and ammonium ion (NH_4_^+^)), nitrate nitrogen (NO_3_^−^-N), nitrite nitrogen (NO_2_^−^-N), active phosphate (PO_4_^3−^-P), active silicate (SiO_3_^2−^-Si), total nitrogen (TN), and total phosphorus (TP). Biological samples included chlorophyll a (Chl *a*) samples, DNA samples, and bacterial density.

### 2.4. Water Quality Analysis

The temperature, turbidity, dissolved oxygen, salinity, and pH of surface seawater were recorded in situ with a YSI EXO^2^ Multiparameter Sonde (YSI Inc., Yellow Springs, OH, USA) at a depth of 50 cm. For the analyses of nutrient content, samples were filtered through glass-fiber filters (Whatman GF/F, 47 mm, 0.68 µm), fixed with chloroform, and then stored at −20 °C. The concentrations of nutrients were analyzed using a continuous flow analyzer (Skalar San++, the Netherlands). For the Chl *a* measurement, 100 mL of seawater was filtered using Whatman GF/F filters after initial filtration through a 200 µm nylon sieve. The filter membrane was wrapped in aluminum foil and stored in a dark environment at −20 °C prior to spectrophotometric analysis. In addition, the samples were fixed with paraformaldehyde (final concentration of 1%) and stored in a freezer at −80 °C before bacterial density determination. After SYBR Green I (1:10,000, *V*/*V*) staining was performed in the dark for 15 min, the bacterial concentration was determined by flow cytometry (FACS Calibur, BD, Franklin Lakes, NJ, USA) [22].

### 2.5. DNA Extraction, PCR Amplification, and Illumina Sequencing

Surface water samples were filtered through a 200 µm nylon sieve to eliminate the interference of zooplankton; following filtration, the samples were passed through a cellulose acetate membrane filter (0.22 µm). The 0.22 µm filters were collected in a centrifuge tube, stored in liquid nitrogen at −196 °C, and then transferred to a –80 °C freezer for storage. Microbial DNA was extracted using the HiPure Soil DNA Kits (Magen, Guangzhou, China) according to the manufacturer’s protocol. The 16S rDNA target region of the ribosomal RNA gene was amplified by PCR (95 °C for 5 min, followed by 30 cycles at 95 °C for 1 min, 60 °C for 1 min, 72 °C for 1 min, and a final extension at 72 °C for 7 min) using V3-V4 gene universal primers 341F (5′-CCTACGGGNGGCWGCAG-3′) and 806R (5′-GGACTACHVGGGTATCTAAT-3′) [23]. For 18S rDNA genes, universal primers 528F (5′-GCGGTAATTCCAGCTCCAA-3′) and 706R (5′-AATCCRAGAATTTCACCTCT-3′) targeting the V4 regions were used for PCR (95 °C for 2 min, followed by 35 cycles at 95 °C for 30 s, 60 °C for 45 s, 72 °C for 90 s, and a final extension at 72 °C for 10 min) [24]. Amplicons were extracted using 2% agarose gels and purified using the AxyPrep DNA Gel Extraction Kit (Axygen Biosciences, Union City, CA, USA) according to the manufacturer’s instructions. Purified amplicons were pooled in equimolar amounts and paired-end sequenced (PE250) on an Illumina platform according to the standard protocols.

### 2.6. Data Processing and Analysis

In order to obtain high-quality clean reads, raw reads were further filtered using FASTP [25] (version 0.18.0). Paired-end clean reads were merged as raw tags using FLASH [26] (version 1.2.11), with a minimum overlap of 10 bp and mismatch error rate of 2%. The clean tags subjected to specific filtering conditions were clustered into operational taxonomic units (OTUs) of ≥97% similarity using the UPARSE [27] (version 9.2.64) pipeline. All chimeric tags were removed using the UCHIME algorithm [28]; finally, effective tags were obtained for further analysis. The tag sequence with the highest abundance was selected as the representative sequence within each cluster. The representative OTU sequences were classified into organisms by a naïve Bayesian model using the RDP classifier [29] (version 2.2) based on the SILVA database [30] (version 132), with a confidence threshold value of 0.8. In order to further investigate the abundance and richness of eukaryotic phytoplankton from the 18S rDNA results of this study, we removed nonalgal OTUs, including Metazoa, Fungi, Apicomplexa, Intramicronucleata, Streptopyta, Postciliodesmatophora, Opalozoa, and unclassified data. In addition, the relative abundance of each taxon was calculated by dividing the sequence number of OTUs of this group by the total sequence number of algae. In addition, chloroplast OTUs (Order: Chloroplast) were removed from 16S rDNA results.

Shannon index was calculated using Qiime software (version 1.7.0) to compare the diversity of the microbial community. The R language Vegan package (version 2.5.3) was used for intragroup Bray–Curtis distance index calculation, Welch’s *t* test, and variance partitioning analysis (VPA). During VPA, we grouped environmental factors. Physical parameters included T, S, pH, TUR, and DO; nutrients included TAN, NO_3_^−^, NO_2_^−^, PO_4_^3−^, and SiO_3_^2−^; and TN and TP were classified as organic matter. The function of the bacterial community was annotated and predicted based on the OTU classification table obtained by using the FAPROTAX (Functional Annotation Prokaryotic Taxa) database [31]. Excel 2013 and Origin8.5 were used to process and plot the data.

The Molecular Ecological Network Analysis (MENA) Pipeline was applied to evaluate the effects of MC on bacterial interspecies interactions using an open-access pipeline available at http://ieg4.rccc.ou.edu/mena [32] (20 March 2021). According to the network topological indexes, the network was visualized by Gephi (version 0.9.2) software. The topological roles of different OTUs could be described by within-module connectivity (Zi) and connectivity among modules (Pi). Zi describes how well a node is connected to other nodes within its own module, and Pi reflects how well a node connects to different modules. Hub and connector taxa are determined by Pi and Zi, with nodes categorized into module hubs (Pi < 0.62 and Zi > 2.5), connectors (Pi > 0.62 and Zi < 2.5), network hubs (Pi > 0.62 and Zi > 2.5), and peripherals (Pi < 0.62 and Zi < 2.5) [33,34]. From an ecological perspective, peripherals represented specialists, whereas module hubs and connectors are similar to generalists, and network hubs are supergeneralists. Network hubs, connectors of the network, may be considered as key species of the communities because they play important roles in maintaining network integrity [35].

## 3. Results

### 3.1. Water Quality Analysis

**Figure 2:**
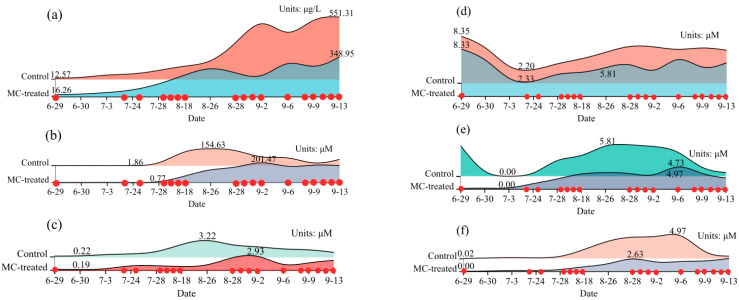
Variation in water quality parameters of the control pond and MC-treated pond through time: (**a**) Chl *a*, (**b**) TAN, (**c**) PO_4_^3−^-P, (**d**) SiO_3_^2−^-Si, (**e**) NO_3_^−^-N, (**f**) NO_2_^−^-N. The red points show the time of MC treatment. The labeled values represent the highest and lowest content.

The variation of water quality parameters in the control pond and MC-treated pond during the investigation was shown in Figure 2. The Chl *a* concentration in the two ponds showed a low and late rising trend, and the Chl *a* content in the control pond fluctuated with time. The nitrate, phosphate, and nitrite content in the water of the MC-treated pond were lower than those in the control pond, and the TAN content was higher than that in the control pond. Temperature, dissolved oxygen, salinity, and pH during the aquaculture process were stable through time, as shown in Table S3.

### 3.2. Microbial Community Composition

**Figure 3:**
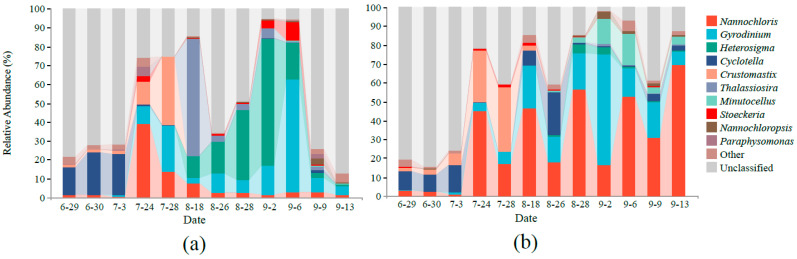
The relative abundance of the ten most abundant phytoplankton genera varied among treatments: (**a**) control pond, (**b**) MC-treated pond.

After quality control, we obtained 2,728,684 effective tags, with an average N50 of 343 bp, by 18S rDNA gene sequencing. After removing nonalgal OTUs, 212 and 222 algal OTUs were obtained in the control pond and MC-treated pond, respectively. Taxonomic analysis of all samples identified 6 phyla, 16 classes, 22 orders, 29 families, 33 genera, and 38 species. The top 10 algal reads at the genus level were defined as the dominant phytoplankton genera. Species abundance in different samples was shown in Figure 3. The phytoplankton in the control pond and the MC-treated pond was dominated by *Cyclotella*, which is diatom, in the early stage (6.29–7.3), which does not rule out the influence of multiple copies [36]. In the middle stage (7.24–8.18), the water was dominated by *Nannochloris* and *Gyrodinium* in both ponds. In the later stage (8.26–9.13), the phytoplankton composition in the control pond was transformed, dominated by *Heterosigma* and *Gyrodinium*, while the phytoplankton composition in the MC-treated pond was maintained as *Nannochloris* and *Gyrodinium*. In addition, *Heterosigma* accounted for the highest proportion (13.09%) in the control pool, which was higher than that in the MC-treated pond (0.92%) (Welch’s *t*-test, *p* value = 0.066); we mainly observed *Heterosigma akashiwo*, an HAB organism. In the MC-treated pond, *Nannochloris* accounted for the highest proportion in the water, with an average relative abundance of 29.9%, which was significantly higher than that of the control pond (Welch’s *t*-test, *p* < 0.05). Moreover, for Dinophyceae, including *Gyrodinium*, *Stoeckeria*, etc., their presence in the control pond (30.02%) was higher than that in the MC-treated pond (25.37%) overall (Welch’s *t*-test, *p* > 0.05). In addition, the microscopic examination results showed that the use of MCs reduced the occurrence of HAB species and was mainly green algae; no toxic algae were detected (unpublished data). For example, in the control pond, the density of *H. akashiwo*, an HAB species, was 1.26 × 10^5^ cells/mL on 2 September, while the MC-treated pond was not detected.

**Figure 4:**
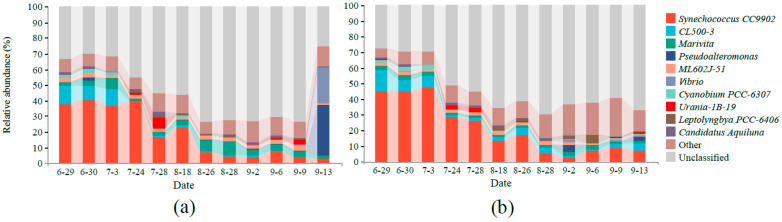
The relative abundance of the ten most abundant bacteria genera varied among treatments: (**a**) control pond, (**b**) MC-treated pond.

After 16S rDNA high-throughput sequencing, 2,851,387 high-quality sequences were obtained from the control pond and the MC-treated pond, with an average N50 of 446 bp. In total, 4351 and 3700 OTUs were obtained at a similarity level of 97%. Taxonomic analysis of all samples identified 51 phyla, 114 classes, 231 orders, 342 families, 579 genera, and 180 species. At the genus level, the relative abundance of the ten most prevalent bacteria was compared in the two ponds (Figure 4), among which *Synechococcus CC9902* demonstrated the highest abundance in the two ponds. The average abundance in the control pond and the MC-treated pond was 18.74% and 21.27% (Welch’s *t*-test, *p* value = 0.71), respectively, which decreased with the shrimp culture time. *Marivita* accounted for 4.15% in the control pond and 2.13% in the MC-treated pond, with a significant difference (Welch’s *t*-test, *p* < 0.05). In addition, the dominant probiotics and opportunistic pathogens at the genus level in aquaculture were analyzed (Table S4). The pathogenic bacteria, such as *Pseudoalteromonas* and *Vibrio*, were more prevalent in the control pond than in the MC-treated pond, with an average of 3.08% and 2.11% in the control pond and 0.66% and 0.29% in the MC-treated pond, respectively (Welch’s *t*-test, both *p* value > 0.05). Compared with the control pond, the proportion of probiotic genera increased, and that of pathogenic bacterial genera decreased in the water of the MC-treated pond. In addition, the results of flow cytometry showed that the average bacterial density in the water of the MC-treated pond was slightly higher (7%) than that in the control pond (Table S3).

### 3.3. Diversity of Microbial Communities

**Figure 5:**
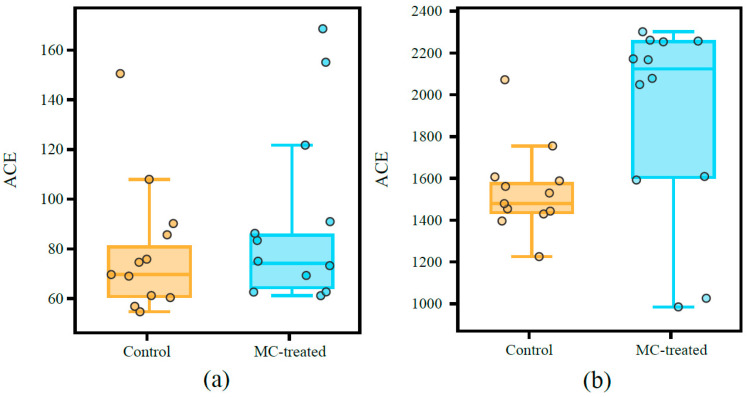
Alpha diversity of OTUs at different times in the control and MC-treated pond: (**a**) phytoplankton, Welch’s *t*-test, *p* value = 0.81; (**b**) bacteria, Welch’s *t*-test, *p* value = 0.03.

After MC treatment, the alpha diversity of eukaryotic phytoplankton, measured as the abundance-based coverage estimate (ACE), was higher than that in the control pond. The median bacterial ACE index in the MC-treated pond was 2119.6, which was significantly higher than that of the control pond, at 1501.4 (Welch’s *t*-test, *p* < 0.05), indicating that the bacterial alpha diversity increased significantly after MC treatment (Figure 5).

**Figure 6:**
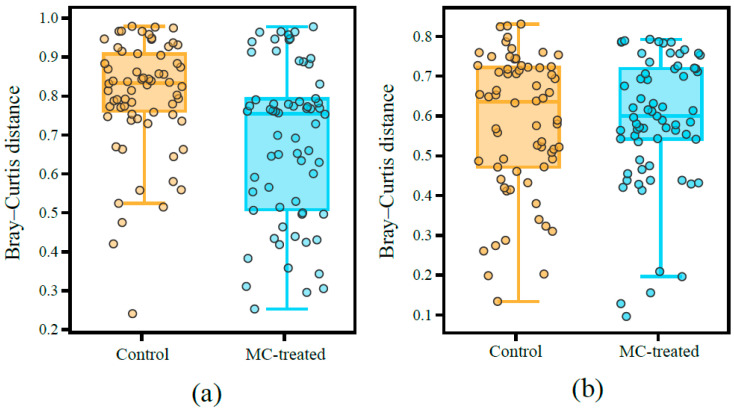
Box plot diagram of mean Bray–Curtis distance at OTU level between different times in the control and MC-treated pond: (**a**) phytoplankton, Welch’s *t*-test, *p* < 0.001; (**b**) bacteria, Welch’s *t*-test, *p* value = 0.29.

Furthermore, the microbial community dissimilarity in beta-diversity among different times based on Bray–Curtis distance was estimated (Figure 6). We found that Bray–Curtis distance of phytoplankton in the control pond within groups at different times was significantly higher than that in the MC pond (Welch’s *t*-test, *p* < 0.001), indicating that the differences of phytoplankton community composition in the control pond varied more significantly over time than that in the MC-treated pond. Meanwhile, the mean Bray–Curtis distance of bacterial community in the control pond at different times was also higher than that in the MC-treated pond.

### 3.4. Network Structure of Microbial Communities

**Figure 7:**
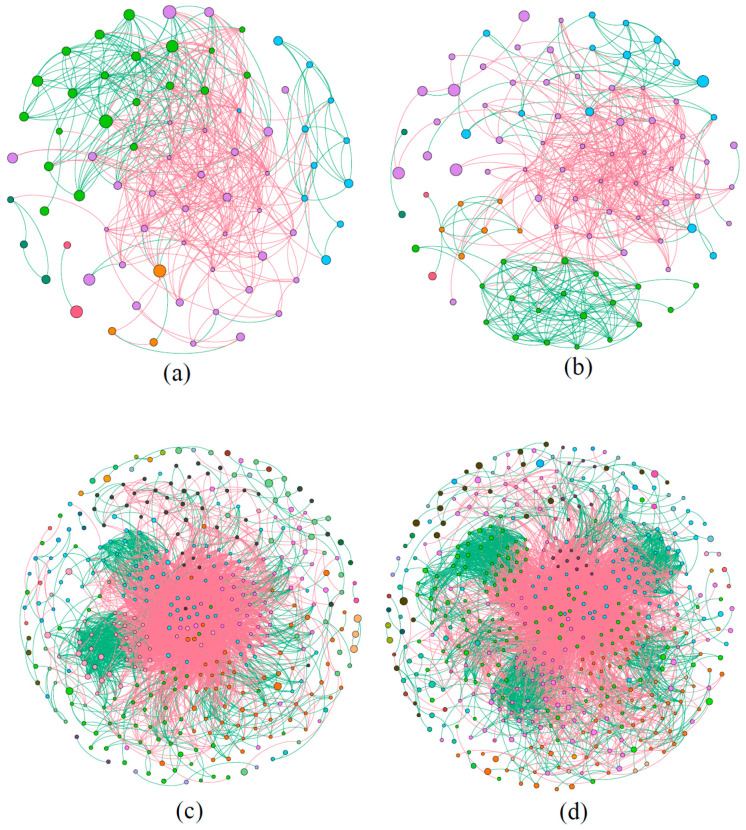
Molecular ecological network of microbial community: (**a**) phytoplankton of control pond, (**b**) phytoplankton of MC-treated pond, (**c**) bacteria of control pond, (**d**) bacteria of MC-treated pond. Each treatment was based on 12 water samples, used to construct an ecological network. Each node represents an OTU, the node size represents the relative abundance of OTUs. The number of nodes and links in the network is an indicator of the scale and complexity of the network. The more nodes and links, the higher the species richness and the complexity of species interaction. The red line indicates a negative correlation, and the green line indicates a positive correlation. The positive and negative correlations in the network can be used to infer the relationships between microorganisms. For example, a positive link represents a niche consistency or symbiotic relationship.

The microbial molecular ecological network analysis suggested that the network topological indexes of phytoplankton and bacteria in water were different between the control pond and the MC-treated pond (Table 1), with the same correlation threshold (0.95). In the phytoplankton molecular ecological network, the number of nodes for the control pond and the MC-treated pond was 74 and 94, respectively, and the number of links was 444 and 501, respectively, which indicated that the MC-treated pond had a more complex network than the control pond (Figure 7a,b). In addition, the average clustering coefficient of the phytoplankton network in the MC-treated pond was lower than that in the control pond, and the average path distance was greater than that in the control pond (Table 1).

Similarly, in the bacterial molecular ecological network (Figure 7c,d), the number of nodes and links of the bacterial molecular ecological network in the MC-treated pond was higher than those in the control pond. In addition, the average clustering coefficient of the bacterial molecular ecological network in the MC-treated pond was lower than that in the control pond, and the average path distance was higher than that in the control pond.

Venn analysis was performed on the nodes (at OTU level) in the phytoplankton molecular ecological network for the control pond and the MC-treated pond (Figure S1). Only 41 nodes were shared by the two ponds, while 45% were unique to the control pond and 56% were unique to the MC-treated pond. Furthermore, in the bacterial molecular ecological network, 222 nodes were common among the control pond and the MC-treated pond. In the control pond, 44% of the nodes were unique, and in the MC-treated pond, 56% were unique.

### 3.5. Key Microbial Populations of Molecular Ecological Network

**Figure 8:**
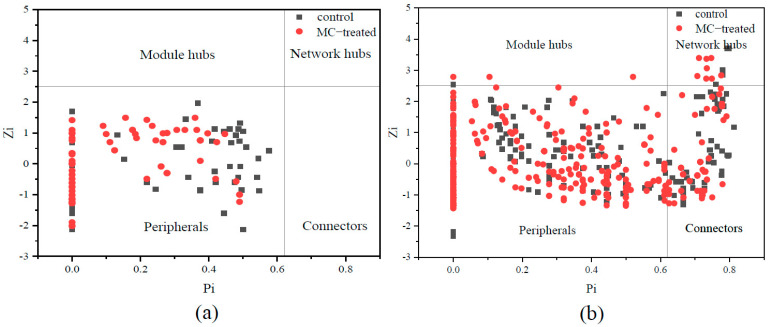
Zi–Pi plot showing the distribution of OTUs based on their topological roles: (**a**) phytoplankton and (**b**) bacteria. Each symbol represents an OTU under the control pond (black) or MC-treated pond (red). The topological role of each OTU was determined according to the scatter plot of within-module connectivity (Zi) and among-module connectivity (Pi). The nodes in a network are divided into the following four subcategories: peripherals (i.e., they have only a few links and almost always to the species within their modules), connectors (i.e., these nodes are highly linked to several modules), module hubs (i.e., they are highly connected to many species in their own modules), and network hubs (i.e., they act as both module hubs and connectors).

Topologically, different OTUs (nodes) play distinct roles in the network. According to the simplified classification used in pollination networks, the topological roles of the OTUs identified in these networks are shown as a Zi–Pi plot in Figure 8. In the phytoplankton molecular ecological network, there were 127 node OTUs in the two ponds, but all nodes were peripherals, without key nodes. In the bacterial molecular ecological network, a total of 675 node OTUs were identified in the control pond and MC-treated pond. The following observations were made: (1) 70.28% of the nodes in the control pond belonged to peripherals, of which 40.81% even had no links at all outside their own modules (i.e., Pi = 0); 83.20% of the nodes of the MC-treated pond belonged to peripherals, of which 33.20% were only linked to some nodes in their own modules. (2) Only one OTU in the control pond was a module hub, while three nodes in the MC-treated pond were module hubs. (3) 28.21% of the nodes in the control pond belonged to connectors, while 14.60% of the nodes of the MC-treated pond were connectors. (4) Five bacterial OTUs in the control pond belonged to the network hubs, namely OTU24, OTU298, OTU482, OTU536, and OTU1046. Moreover, there were nine bacterial OTUs as network hubs in the MC-treated pond, namely OTU545, OTU600, OTU705, OTU781, OTU1004, OTU1081, OTU1114, OTU1131, and OTU1165.

## 4. Discussion

### 4.1. Effect of MC on Phytoplankton Community Structure in Aquaculture Water

As an important primary producer in aquaculture water, phytoplankton plays a fundamental role in material circulation and water self-purification [37]. In addition, most marine phytoplankton are unicellular organisms and sensitive to environmental changes [38]. The variations in phytoplankton can reflect not only the health state of the aquaculture water ecosystem, but also the structure and function of the aquatic ecosystem [39].

While the surface of MC is positively charged, the surface of phytoplankton is negatively charged [14]; thus, MC can easily cause flocculation and settling after collision and contact with phytoplankton. In addition, MC could stimulate HAB organisms to generate large amounts of reactive oxygen species, inhibiting growth of remaining cells [14]. The use of MC had a certain impact on the phytoplankton community in aquaculture water. The control pond was mainly dominated by dinoflagellate and *H. akashiwo*, which may have adverse effects on the growth of *L. vannamei*, such as slow growth, enteritis, liver disease, and even death [40]. Moreover, *H. akashiwo* bloomed at the end of August and the beginning of September in the control pond, resulting in drastic changes in water color. However, there was no dramatic change in water color in the MC-treated pond, and green algae, such as *Nannochloris*, accounted for a high proportion. *Nannochloris* has high lipid, protein, and linoleic acid content, and it is widely used for biodiesel [41], phytoplankton bait [42], environmental protection [43], and biological medicinal purposes [44]. In addition, it can improve the survival rate and yield of fish and shrimp when used as aquatic bait [42]. In this experiment, it was found that the average body length and weight of shrimp in the MC-treated pond were significantly higher than those in the control pond (Welch’s *t*-test, *p* < 0.05), after the harvest of *L. vannamei* on 17 October 2020 (Figure S2). Note that the use of MC maintained a better phytoplankton composition in the process of *L. vannamei* culture, which promoted the high yield of shrimp.

In addition, the use of MC improved the alpha diversity of phytoplankton (Figure 5a). Studies suggest that the higher the diversity index of phytoplankton in the water body, the more complex the algae, which is conducive to the stability of the aquaculture water environment and has a high buffer capacity against changes of external factors; it also results in a high buffer capacity against changes in external factors [45]. In this study, the Bray–Curtis distance for phytoplankton in the control pond at different time samples was significantly higher than that in the MC-treated pond (Figure 6a), indicating that the phytoplankton composition in the control pond fluctuated greatly in time series, while the phytoplankton community in the MC-treated pond was more stable over time.

The MENA indicated that the use of MC improved the number of nodes, links, as well as average path distance of the phytoplankton community, and it reduced the average clustering coefficient (Table 1). The number of nodes and links in the network is an indicator of the scale and complexity of the network. In this study, the species richness and species interaction complexity in the phytoplankton molecular ecological network of the MC-treated pond were higher than those of the control pond. Previous studies have suggested that a short average path distance of the network indicates that all the nodes in the network are closer to one another, which means that the transmission efficiency of information, energy, and substances among species is higher [46], and the response speed of the microbial community is faster [47]. Compared with the MC-treated pond, the average path distance of phytoplankton network in the control pond was shorter, indicating that when the environment was disturbed, the response speed of phytoplankton in the control pond was faster, and the phytoplankton community was more likely to change. Although the MC was treated many times, the response speed of phytoplankton in the MC-treated pond was slow, consistent with the results of Xiong et al., who found that environmental disturbances reduced the speed of change in the microbial community structure [48]. In addition, a higher average clustering coefficient indicates that microorganisms are more sensitive to the interference of external environmental factors [49]. In this study, the average clustering coefficient of phytoplankton in the control pond was higher than that in the MC-treated pond, indicating that the phytoplankton in the control pond were highly sensitive to the external environment and responded quickly, while the anti-interference ability of the phytoplankton community in the MC-treated pond was stronger. Moreover, the positive and negative correlations in the network can be used to infer the relationships between microorganisms. A positive correlation represents a niche consistency or symbiotic relationship, such as the building of biofilms or the exchange of metabolites between microorganisms. In contrast, a negative correlation indicates competition or a predator–prey relationship [50]. The phytoplankton molecular ecological networks constructed in this study were dominated by a negative correlation, indicating that the competition between phytoplankton in aquaculture water was greater than the cooperation. In summary, the phytoplankton community in the MC-treated pond had high species richness and complexity, a more stable community, and higher anti-interference ability, which are beneficial for the growth of shrimp, while the phytoplankton interaction in the control pond was sparse and sensitive to the environment. In conclusion, the composition of phytoplankton in MC-treated pond was better, which was closely related to the regulation of MC. On the one hand, the MC can directly inhibit the growth of HAB species. On the other hand, the MC maintained the stability of the whole phytoplankton community, inhibiting the mass reproduction of HAB species from the interspecific competition.

**Figure 9:**
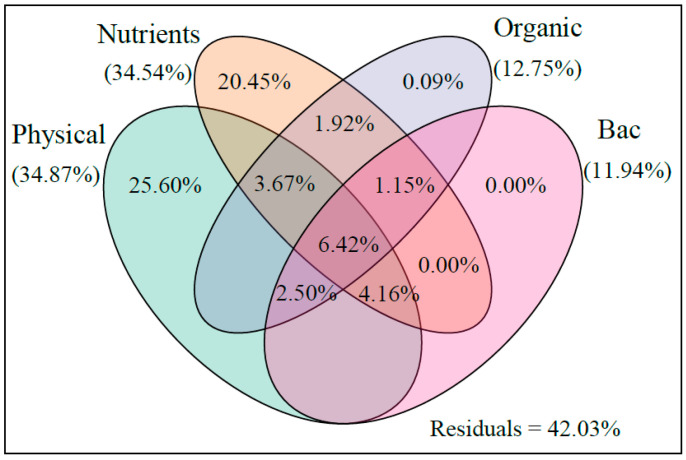
Variation partitioning analysis of the relative contributions of physical parameters, nutrients, organic matter, and bacteria density variables to phytoplankton community structure variation. Physical parameters included T, S, pH, TUR, and DO; nutrients included TAN, NO_3_^−^, NO_2_^−^, PO_4_^3−^, and SiO_3_^2−^; and TN and TP were classified as organic matter. Each number represents the biological variation partitioned into the relative effects of each factor or a combination of factors. The values < 0 are not shown.

Furthermore, VPA was used to analyze the contribution of physical parameters, nutrients, organic matter, and bacteria density variables to phytoplankton community variation. As illustrated with a modified variation partitioning diagram (Figure 9), the complete set of all variables explained 57.97% of the variation in the phytoplankton communities, with physical properties and nutrients clearly displaying the greatest contributions. The changes in physical parameters, including DO and salinity, were essentially the same in the two ponds, and they were not the main factors causing the differences in the phytoplankton communities between the two ponds. As one of the factors most associated with phytoplankton growth, changes in nutrients significantly affect phytoplankton communities. Different phytoplankton responded inconsistently to variations in the N and P concentrations. Variations in the N and P concentrations could lead to a sharp rise in some species and a sharp decline in other species [51]. In this study, due to the adsorption of phosphate by MC [16], the phosphate content in the MC-treated pond fluctuated less than that in the control pond through time (Figure 2). Similarly, the change in nitrite content in the MC-treated pond over time was lower than that in the control pond. When the fluctuation of nutrients is low, the stability of the phytoplankton community is maintained.

### 4.2. Effect of MC on Bacteria Community Structure in Aquaculture Water

Bacteria play an important role in nutrient circulation, water quality maintenance, and the health of cultured animals [52]. In contrast to terrestrial animals, aquatic animals and microorganisms share the same water environment in the aquaculture ecosystem [53]. Therefore, aquatic animals are highly vulnerable to the changes in the surrounding bacterial community [54]. Maintaining the stability of the bacterial community is very important for the growth of cultured organisms.

By analyzing the bacterial composition, we found that probiotic and pathogenic bacterial genera were different in water samples collected at different times (Table S4). Overall, the proportion of pathogenic bacteria decreased with the addition of MC, accompanied by an increase in probiotics. Among the pathogens, *Vibrio* and *Pseudoalteromonas* accounted for a relatively high proportion, and their proportion in the control pond was large and fluctuated significantly over time, while the proportion in the MC-treated pond was small. *Vibrio* and *Pseudoalteromonas* are considered opportunistic pathogenic bacteria [53,55], and opportunistic pathogenic bacteria are pervasive in seawater and become dominant when the aquaculture water quality deteriorates [7,56]. In fact, the high mortality of shrimp is attributed to the proliferation of opportunistic pathogenic bacteria [57,58,59]. In the control pond, the sudden increase in opportunistic pathogenic bacterial content may have an adverse impact on the growth of shrimp. It is speculated that the low pathogens content in MC-treated pond may be the inhibitory effect of MC on pathogens, for which the main mechanisms are flocculation and oxidative damage (unpublished data).

**Figure 10:**
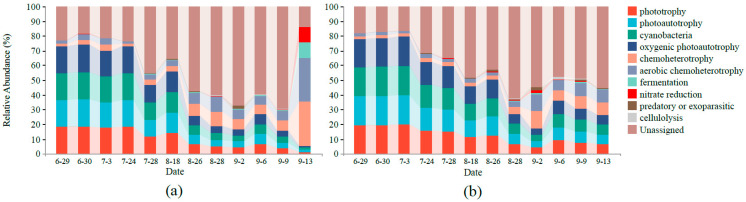
Prediction of the bacterial functional profiles of the core microbiome in the two ponds using FAPROTAX software: (**a**) control pond; (**b**) MC-treated pond.

Moreover, the bacterial function was predicted using FAPROTAX (Figure 10). The photoautotrophic function (including phototrophy, photoautotrophy, cyanobacteria, and oxygenic photoautotrophy) accounted for a relatively high proportion in the two ponds, and the proportion gradually decreased over time. Secondly, the relative abundance of the chemoheterotrophy function (including chemoheterotrophy and aerobic chemoheterotrophy) was high, and the proportion gradually increased over time. In addition, compared with the MC-treated pond, the function of the bacterial community in the control pond fluctuated greatly. From 29 June to 13 September, the photoautotrophic function of bacterial community in the control pond decreased from 72.8% to 5.5%, and the chemoheterotrophy function increased from 3.9% to 59.4%. In the MC-treated pond, the photoautotrophic function decreased from 77.9% to 26.0%, and the chemoheterotrophy function decreased from 3.3% to 16.8%. The alpha diversity results showed that the diversity of bacteria in the MC-treated pond was higher than that in the control pond (Figure 5b). Studies have shown that there is a positive relationship between microbial taxonomic diversity and ecosystem function [60,61,62,63], and increasing community diversity can improve ecosystem stability [64,65,66]. Moreover, highly diverse communities in aquatic ecosystems are conducive to the stability of microbial communities under environmental changes [67], which helps to maintain the function of ecosystems [68]. In this study, the alpha diversity of the bacterial community in the water of the MC-treated pond was high, and the bacterial function fluctuated little over time, which was consistent with previous research results. In addition, the average Bray–Curtis distance of the bacterial community in the control pond at different times was higher than that in the MC-treated pond (Figure 6b), which also indicated that the bacterial community structure in the control pond fluctuated significantly over time and had poor stability.

Regarding the bacterial molecular ecological networks, the number of nodes and links for that of the MC-treated pond were higher than those in the control pond; moreover, the average clustering coefficient was lower, and the average path distance was longer. This indicates that the species richness and species interaction complexity of bacteria in the MC-treated pond were higher than those in the control pond, the response speed of bacteria in the MC-treated pond was low, and disturbances in the external environment would not affect the whole bacterial ecological network in a short time. Another possible reason for the low content of pathogens in the MC-treated pond was that the appropriate MC could maintain the stability of the bacterial community structure in the aquaculture water. Thus, the possibility of a large-scale reproduction of pathogenic bacteria would be reduced.

Key species in the molecular ecological network are those that play a critical role in the ecological community structure and ecosystem stability, directly determining the structure and function of the ecosystem. If these species are removed from the ecosystem, this may lead to fundamental changes in the ecosystem, and other species will be directly or indirectly affected [69]. In this study, the use of MC changed the number of key bacterial nodes in aquaculture water and the nodes of molecular ecological network (Figure S1). It was indicated that MC regulated the key species of the microbial network, causing changes in the molecular ecological network’s structure and function. Moreover, the ecological functions of key species in the control pond and MC-treated pond were different. In the control pond, the network hubs OTU24 and OTU482 were Rhodobacteraceae, belonging to Proteobacteria, which are important heterotrophic bacteria in the marine environment [70] and have symbiotic or pathogenic effects on phytoplankton and macroalgae [71], which prove their impact on phytoplankton in the marine ecosystem. In this study, OTU24 and OTU482 were the key nodes in the control pond, indicating that they also played an important role in the mariculture ecosystem. In addition, OTU536, the network hub of the control pond, belonged to Bradymonadales, which has a strong ability to prey on other bacteria [72] and can store nutrients in cells in the form of polymers during the predation process. Considering their extensive predation ability, it is speculated that Bradymonadales may regulate the construction of the microbial community and element cycle in aquaculture environments. In the MC-treated pond, OTU545 belonged to Cryomorphaceae. The species of this family play an important role in secondary production in the aquatic ecosystem, and they are usually found in regions with relatively high organic carbon content [73]. OTU600 belonged to Pseudoalteromonadaceae. Many species of this family produce a variety of primary and secondary metabolites, with antibacterial and antiviral properties [74]. Due to their various metabolic abilities, it is speculated that these species have strong adaptability in aquaculture water and play an important ecological role in coping with environmental changes. OTU781 was a Bacteriovoracaceae, which can parasitize and lyse common pathogenic bacteria such as *Vibrio* in mariculture. These species can change the structure of the environmental microecological community [75], prevent and control aquaculture biological bacterial diseases [76], and improve the survival rate of aquaculture individuals [75]; this family is an important regulator of the microbial ecological balance. In conclusion, the use of MC affected the key species of the bacterial community network in aquaculture water and changed the structure of the microbial molecular ecological network, subsequently changing the structure and function of the bacterial community.

**Figure 11:**
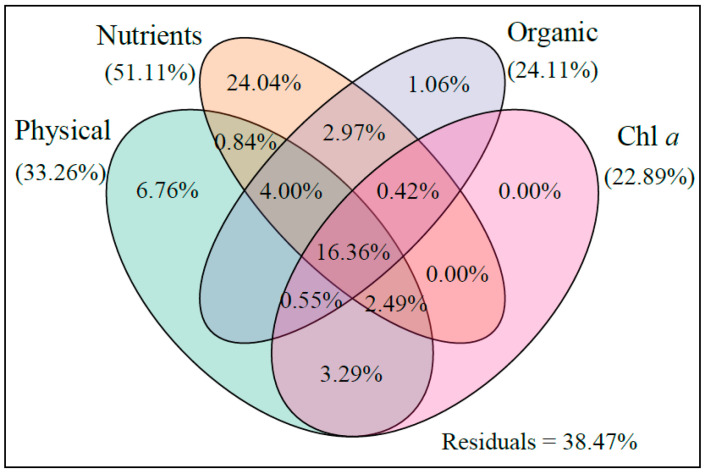
The influence of physical parameters, nutrients, organic matter, and Chl *a* features on the bacterial community structure estimated via VPA. Physical parameters included T, S, pH, TUR, and DO; nutrients included TAN, NO_3_^−^, NO_2_^−^, PO_4_^3−^, and SiO_3_^2−^; and organic matter included TN and TP. The percentages represent the variance explained. The values < 0 are not shown.

The results of environmental factor analysis (Figure 11) showed that the variance in nutrient content explained 51.11% of the variation in the bacterial communities. Bacterial growth also requires nutrients [77]. The MC regulated the nutrients in the water [16,78], which also maintained the stability of the bacterial community. In addition, physical parameters (33.26%), organic matter (24.11%), and Chl *a* (22.89%) could also describe the bacterial variance. The changes in the physical parameters were essentially the same in the two ponds; they were not the main factors causing the differences in the bacterial communities. Organic matter quality is partly responsible for changes in bacterial community composition [79]. MC has a certain regulatory effect on organic matter, which can also indirectly control the composition of the bacterial community [15]. Chl *a* was an important environmental factor affecting the bacterial community, indicating that some bacteria may have a symbiotic relationship with phytoplankton [80], and the regulatory effect of MC can not only stabilize algae but also indirectly maintain the stability of the bacterial community.

## 5. Conclusions

High-throughput sequencing technology and random matrix network construction theory were used to analyze the effects of MC on the microbial community structure and the interaction network between different species at the pond scale. The use of MC maintained a microbial community composition that was favorable for shrimp growth, mainly composed of green algae, a lower content of pathogens, and a higher content of probiotics. Furthermore, the diversity and stability of phytoplankton and bacteria in the MC-treated pond were higher than those in the control pond, and the molecular ecological network of the MC-treated pond had high anti-environmental interference ability. MC can not only directly affect the key species of the microbial molecular ecological network but also indirectly affect water-quality parameters, thus changing the microbial composition and community structure. These findings provide novel information concerning the improvement of microflora in aquaculture environments, which is helpful to maintain the health and high yield of shrimp.

## Figures and Tables

**Table 1:** The network topological indexes in the microbial molecular ecological network of aquaculture water.

Network Indexes	Phytoplankton		Bacteria		Explanation
	Control	MC-Treated	Control	MC-Treated	
Total nodes	74	94	397	500	Network scale
Total links	444	501	3791	4590	Species interaction complexity
Average clustering coefficient	0.385	0.375	0.253	0.224	Node connection degree
Average path distance	2.464	2.833	3.150	3.315	Response speed
Positive link percentage (%)	36.26	38.52	23.48	30.98	Cooperation and mutualisms

## Data Availability

Data are available upon request; please contact the contributing authors.

## Supplementary Materials

The following are available online at https://www.mdpi.com/article/10.3390/ijerph182111569/s1, Table S1: Composition of different elements in kaolin clay, Table S2: Time and dosage of MC in *L. vannamei* culture, Table S3: Environmental factors of all the data points, Table S4: List of designated identified bacterial genera (pathogenic genera and probiotic genera) in *L. vannamei* aquaculture ponds, Figure S1: Venn diagram of unique and common nodes of microbial molecular ecological network in the control and MC treated pond, Figure S2: Monitoring of body length and weight of shrimp during growth.
