# Supplementary material for: Healthier Communities of Phytoplankton and Bacteria Achieved via the Application of Modified Clay in Shrimp Aquaculture Ponds
Source: Int J Environ Res Public Health. 2021 Nov 4;18(21):11569. doi: 10.3390/ijerph182111569 (PMC8583407; doi:10.3390/ijerph182111569)
Supplement: Supplementary file 1 [file ijerph-18-11569-s001.zip › ijerph-1406657-supplementary.pdf]

International Journal of
Environmental Research and Public Health

MDPI

Healthier Communities of Phytoplankton and Bacteria Achieved via the Application of Modified Clay in Shrimp Aquaculture Ponds

Table S1. Composition of different elements in kaolin clay (%, represented in oxidese) (Wenbin Jiang, Unpublished data).

**Analyte**	SiO_2	Al_2O_3	K_2O	Fe_2O_3	MgO	CaO
**Result**	48.01±0.3	31.78±0.18	2.03±0.02	1.07±0.01	0.22±0	0.13±0
**Analyte**	Na_2O	TiO_2	MnO	P_2O_5	SO_3	LOI
**Result**	0.02±0	0.07±0	0.06±0	0.08±0	0.4±0	16.12±0.51

Table S2. Time and dosage of MC (modified clay) in *L. vannamei* culture. The specific use time depended on the on-site concentration of phytoplankton or pathogen and water quality characteristics. On June 29, high concentration MC Ⅰ was used to control the microorganisms, nutrients and organic matter in the water before releasing shrimp seedlings. Subsequently, due to the continuous feeding of bait, biological excretion and microalgae death, various nutrients (especially organic matter) and phytoplankton were high in the aquaculture water over time. The MC of low concentration and repeated treatment was used to control the microorganisms and water quality characteristics.

**Date**	**Dosage of MC (kg)**		**Concentration of MC (g/L)**
	**MC Ⅰ**	**MC Ⅱ**	
6-29	37		0.037
7-17	2.5		0.0025
7-25	2.5		0.0025
7-29	3		0.003
7-30	3		0.003
8-3	3		0.003
8-18	3		0.003
8-28	3		0.003
8-31		3	0.003
9-1		3	0.003
9-6		3	0.003
9-8		3	0.003
9-11		3	0.003
9-12		3	0.003
9-13		1.4	0.0014

Table S3. Environmental factors of all the data points. Bac stands for bacterial density, unit: cells/ml; chla: μg/L; T: ℃; DO: mg/L; S: ‰; TUR: NTU; NO_3^-, PO_4^{3-}, NO_2^-, TAN, SiO_3^{2-}, TN, TP: μM.

**Sample**	**Bac**	**Chla**	**T**	**DO**	**S**	**pH**	**TUR**	**NO_3^-**	**PO_4^{3-}**	**NO_2^-**	**TAN**	**SiO_3^{2-}**	**TN**	**TP**
**Control**														
6-29	2.39E+07	12.57	26.97	6.40	36.72	8.40	5.64	5.40	0.23	0.02	2.67	8.35	123.08	3.63
6-30	5.74E+07	14.43	26.26	5.56	36.90	8.37	5.61	0.00	0.22	0.09	3.18	6.78	122.55	2.43
7-3	4.81E+07	44.33	25.50	6.51	37.45	8.32	4.81	0.00	0.51	0.19	2.96	2.27	129.99	2.49
7-24	9.83E+07	44.63	25.80	7.40	35.95	8.24	6.78	0.01	0.58	0.09	1.86	2.20	111.51	4.82
7-28	7.04E+07	78.31	27.71	7.59	36.14	8.25	6.25	3.33	0.76	0.16	5.13	3.60	162.06	6.75
8-18	2.32E+07	122.08	29.94	6.94	31.87	8.15	10.01	3.23	2.24	1.58	143.53	4.28		
8-26	4.25E+07	137.40	25.34	7.00	31.01	8.18	11.94	5.81	3.22	3.23	154.63	5.52	354.19	3.76
8-28	4.99E+07	316.05	24.70	8.85	30.04	8.30	9.37	5.57	1.93	3.94	146.54	6.79	347.33	4.04
9-2	4.40E+07	523.19	26.40	8.41	31.33	8.49	15.55	5.31	1.86	3.67	59.46	6.55	347.23	18.07
9-6	5.02E+07	342.34	24.41	8.19	30.89	8.21	20.12	4.79	1.34	4.97	89.51	5.78		
9-9	5.14E+07	557.82	25.22	9.43	31.16	8.46	24.89	1.04	1.37	0.50	3.32	6.50	428.50	13.10
9-13	3.66E+07	551.31	23.64	5.49	31.63	7.74	16.57	0.66	0.85	0.43	56.86	5.87	536.76	6.57

**MC-treated**														
6-29	5.29E+07	16.26	27.67	6.16	36.70	8.19	5.78	0.08	0.27	0.00	2.53	8.33	146.36	2.42
6-30	6.28E+07	20.87	26.37	5.48	36.82	8.20	6.94	0.18	0.19	0.02	2.64	6.88	137.27	3.26
7-3	5.76E+07	34.28	25.10	6.49	37.55	8.13	6.05	0.00	0.24	0.16	4.19	2.39	118.33	2.65
7-24	8.58E+07	53.78	25.51	7.63	35.82	8.16	7.49	0.97	0.95	0.07	1.46	2.33	100.86	5.09
7-28	9.43E+07	107.08	27.53	7.70	36.07	8.26	9.45	1.70	0.87	0.23	0.77	4.16	184.94	7.68
8-18	5.46E+07	188.74	29.95	7.14	30.39	8.19	19.35	2.82	0.69	0.83	58.46	4.04	120.42	3.56
8-26	6.06E+07	267.14	25.22	7.19	29.83	8.3	13.58	2.88	0.74	1.15	89.47	5.03	357.46	6.96
8-28	5.50E+07	209.32	24.63	8.25	28.22	8.19	16.81	2.89	2.36	2.63	129.69	6.17	397.16	5.38
9-2	3.25E+07	141.74	26.30	7.48	30.24	8.23	15.59	1.88	2.93	1.35	201.47	3.44	320.93	11.95
9-6	2.54E+07	342.83	24.41	8.10	29.28	8.30	21.30	4.73	0.56	1.80	128.73	7.77		
9-9	2.33E+07	228.10	25.15	7.59	30.32	8.20	27.30	2.48	1.40	1.73	164.69	4.30	429.11	6.39
9-13	3.16E+07	348.95	23.22	8.43	30.84	8.38	22.35	1.94	1.77	2.25	152.46	5.90	451.13	11.53

Table S4. List of designated identified bacterial genera (Pathogenic genera and Probiotic genera) in *L. vannamei* aquaculture ponds.

The genera of bacteria	Average relative abundance (%)	
**Pathogenic genera**	**Control**	**MC-treated**
Vibrio	2.105767	0.2884
Pseudoalteromonas	3.080933	0.664142
Escherichia	0.004399	0.001315
Pseudomonas	0.000747	0.000583
**sum**	**5.191846**	**0.95444**
**Probiotic genera**	**Control**	**MC-treated**
Bacillus	0.000312	0.000352
Bdellovibrio	0.003338	0.005188
Lactococcus	0.000238	0.00019
Streptococcus	0.000185	0
Streptomyces	0.000466	0
Enterococcus	0.000185	0.000385
Bacteroides	0.000842	0.000554
Lactobacillus	0.001805	0.003547
**sum**	**0.00737**	**0.010217**

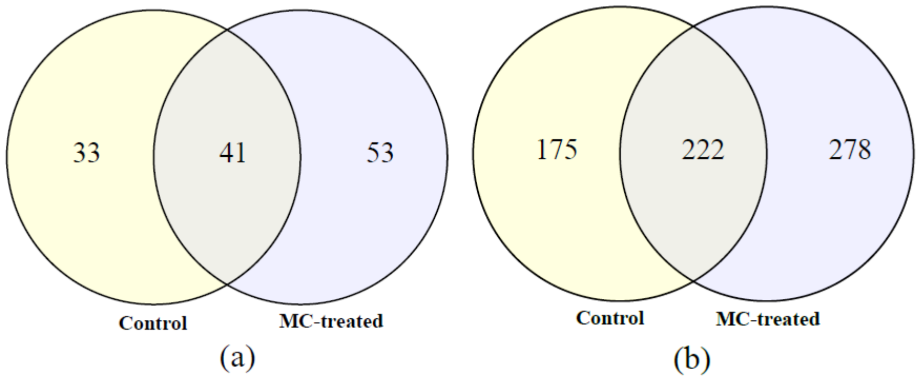

Figure S1. Venn diagram of unique and common nodes of microbial molecular ecological network in the control and MC-treated pond: (a) phytoplankton, (b) bacteria.

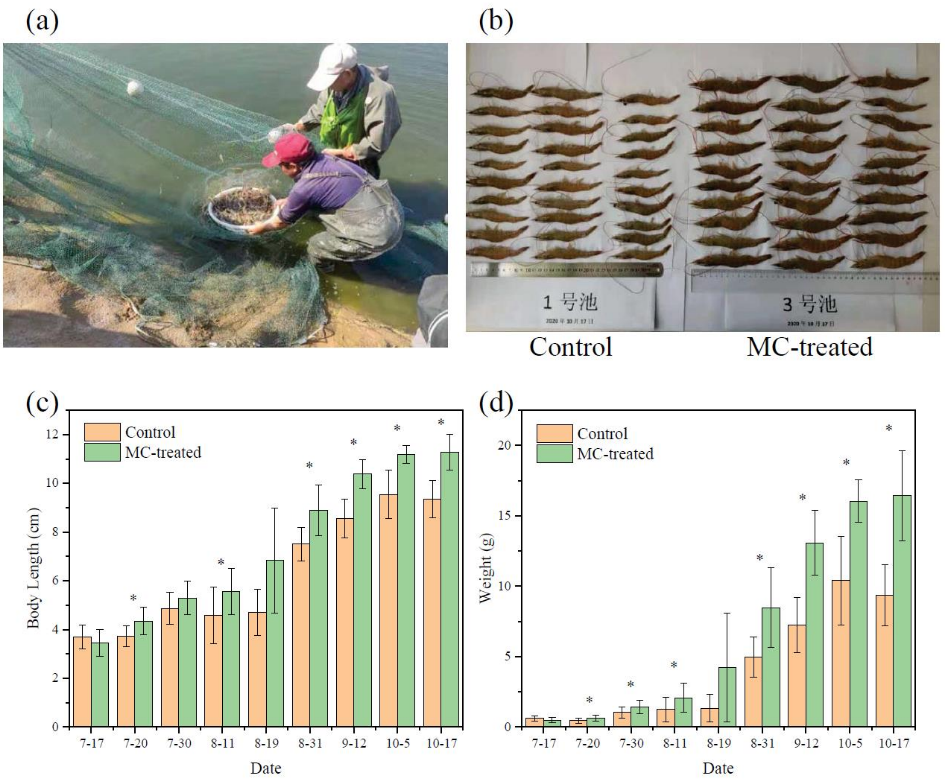

Figure S2. Monitoring of body length and weight of shrimp during growth. On June 30, 2020, about 50000 *L. vannamei* fries were put into control pond and MC-treated pond respectively, with an average body length of 1.6 cm/shrimp and an average weight of 0.035 g/shrimp. On October 17, 2020, shrimp was harvested in control pond and MC-treated pond (a, b). 30 shrimp were randomly selected from each of the two ponds for determination of body length (c) and weight (d) of shrimp, asterisks indicate significant difference (*, $p < 0.05$; Welch's *t*-test).
